# A framework for translational therapy development in deep brain stimulation

**DOI:** 10.1038/s41531-024-00829-5

**Published:** 2024-11-08

**Authors:** Jiazhi Chen, Jens Volkmann, Chi Wang Ip

**Affiliations:** Department of Neurology, University Hospital of Würzburg, Josef-Schneider-Straße 11, Würzburg, Germany

**Keywords:** Motor control, Diseases of the nervous system, Cellular neuroscience

## Abstract

Deep brain stimulation (DBS) is an established treatment for motor disorders like Parkinson’s disease, but its mechanisms and effects on neurons and networks are not fully understood, limiting research-driven progress. This review presents a framework that combines neurophysiological insights and translational research to enhance DBS therapy, emphasizing biomarkers, device technology, and symptom-specific neuromodulation. It also examines the role of animal research in improving DBS, while acknowledging challenges in clinical translation.

## Introduction

Invasive neuromodulation techniques currently encompass deep brain stimulation (DBS)^1^, motor cortex stimulation (MCS)^2^, and spinal cord stimulation (SCS)^3^. In particular, DBS has gained substantial recognition and widespread adoption, and has emerged as a second-line treatment option for patients with Parkinson’s disease (PD), dystonia, or essential tremor (ET) who are refractory to pharmacological interventions^4–6^.

Preclinical studies employing animal models are indispensable for exploring novel targets and indications, elucidating the underlying mechanisms, and bridging the gap between theoretical concepts and practical applications^7^. Since the pioneering implantation of a DBS electrode in the ventral intermediate nucleus (Vim) for tremor in a PD patient in 1987^8^, DBS has evolved over three decades from a reversible lesioning alternative to a complex brain interface technology allowing to interact with defective neuronal circuit activity in specific spatial and temporal domains. Recent technological advances primarily aim to optimize therapeutic efficacy, minimize adverse effects, and reduce stimulator energy consumption. To date, substantial efforts in basic science have been dedicated to advancing DBS in PD. In this review, we examine current clinical challenges in DBS implementation and the corresponding translational methodologies aimed at improving DBS methods. We highlight the accomplishments of model studies and delineate the translational trajectory.

Building upon innovative concepts such as ‘cell type-specific’ targeting for symptom-specific stimulation^9^ and early stimulation aimed at disease-modifying effects^10^, our review explores fundamental discoveries relevant for clinical neuromodulation. We highlight the importance of backtranslating methodologies commonly employed in clinical practice to experimental investigations in appropriate animal models, thus facilitating mutual validation of stimulation mechanisms in a preclinical context. While our focus remains on the application of DBS in PD, it is essential to acknowledge that the translational strategies discussed are not limited solely to PD. Rather, they possess the potential for broader optimization and innovation across various other movement disorders.

## Established invasive neuromodulation therapy in movement disorders

### DBS in PD

In the early stages of PD diagnosis—characterized by motor symptoms—the objective is to compensate for the striatal dopamine deficiency resulting from neurodegeneration of the nigrostriatal dopamine pathway. Standard treatment regimens include administration of medication such as levodopa (L-dopa), dopamine agonists, and monoamine oxidase-B inhibitors^11^. With disease progression the residual dopaminergic terminals are no longer capable of maintaining striatal dopamine homeostasis, which together with postsynaptic sensitization phenomena, causes increasing fluctuations between hypo- and hyperdopaminergic behaviors^11,12^. DBS of the globus pallidus internus (GPi) or the subthalamic nucleus (STN) were originally introduced for the treatment of motor fluctuations and dyskinesia in advanced PD, but more recently have been proposed for extending the ‘honeymoon period’ in earlier disease stages^13^ and for treating neuropsychiatric symptoms in PD associated with drug induced dopaminergic sensitization^14^. These ‘preventive’ indications, however, are not generally accepted in the staged therapeutic approach to PD and await confirmation by randomized, controlled clinical trials.

DBS for motor fluctuations in advanced PD has shown to be safe and effective. Currently, the STN and the GPi are approved for these indications, with both targets being differently chosen by physicians in Europe and North America^15^. In Europe, STN-DBS is generally preferred due to a stronger anti-akinetic effect and more pronounced drug savings after surgery^16^, while GPi-DBS is still largely practiced in North America due to a less complicated postoperative adjustment period and less risk of deterioration in gait and balance^15,17^. A recent meta-analysis revealed a change in the United Parkinson’s Disease Rating Scale (UPDRS)-III motor score (mean follow-up 13 months) after STN-DBS (50.5% reduction compared to baseline) and GPi-DBS (29.8% reduction)^18^. However, neither meta-analyses nor randomized, controlled clinical trials can address these differences in practice, which also reflect different approaches to the pharmacological management of PD, different escalation strategies within the course of disease and different surgical inclusion criteria for surgery.

### DBS in dystonia

Dystonia is a complex clinical syndrome characterized by sustained or intermittent muscle contractions that cause abnormal, often repetitive movements, postures, or both^19^. The clinical and pathogenetic heterogeneity of dystonia can pose challenges in decision-making for DBS interventions tailored to specific dystonia patients^20^. Based on current knowledge, high-frequency stimulation of the GPi is the most effective DBS treatment for dystonia^20^. The effectiveness and relative safety of GPi-DBS for dystonia have been extensively reported in studies involving selected and medication-refractory dystonia patients, including those with primary generalized or segmental dystonia^21–24^, cervical dystonia^25^, and tardive dystonia^26,27^. A meta-analysis summarizing the efficacy of GPi-DBS in early-onset dystonia showed an improvement of 60.6% in the motor score and 57.5% in the disability score (Burke-Fahn-Marsden Dystonia Rating Scale)^28^. Furthermore, STN-DBS has been suggested as a viable alternative option for cervical dystonia with less risk of inducing Parkinsonian symptoms by high-frequency stimulation. A direct comparison of STN- vs. GPi-DBS for dystonia in a larger randomized clinical trial is pending, but clinical observations suggest, that the STN target could be better suited for fixed dystonias, whereas GPi-DBS could be more effective for dystonic movements^29–32^.

### DBS in ET

High-frequency Vim-DBS for tremor was first reported in 1980, resulting in suppression of severe intention tremor of the upper limb after Vim-DBS^33^. This study paved the way for DBS interventions targeting drug-resistant tremors and other movement disorders. Gradually, Vim-DBS replaced traditional thalamotomy^34,35^ for the treatment of drug-resistant tremors. After decades of clinical validation, Vim-DBS for ET is now established and its effectiveness and safety have been proven^36–38^. A recent review reported that unilateral Vim-DBS reduced tremor by 53–63%, while bilateral Vim-DBS led to better tremor improvement, ranging from 66–78%^39^. An emerging DBS target—the posterior subthalamic area—has been found to be effective in the treatment of ET. High-frequency stimulation of this region resulted in a 64% improvement in tremor in a randomized, double-blind, crossover phase study^40^ and an 89% improvement in an earlier non-randomized study^41^.

## Historical translation

The current application of STN-DBS for PD is based on translational studies conducted with a 1-methyl-4-phenyl-1,2,3,6-tetrahydropyridine (MPTP)-treated non-human primate (NHP) model^42^. This translational process first included uncovering pathophysiological changes in the basal ganglia (BG) in this model^43–47^. Subsequently, lesion-based surgery^48^ and high-frequency STN-DBS were applied to this model^49^ and further validation of STN-DBS was successful in PD patients^50^.

**Fig. 1:**
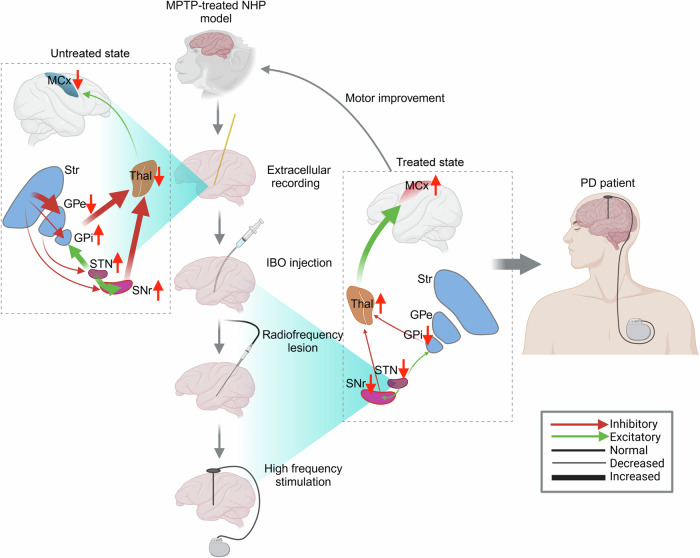
Translational process of STN-DBS. In a parkinsonian-rendered MPTP-treated NHP model^42^, extracellular recordings uncovered an increased tonic discharge pattern in the STN and its downstream nuclei (the GPi), but a decreased firing rate in the GPe^51^. This observation corresponds to the ‘motor circuit’ model^43,47^, where excessive inhibition of the GPe within the ‘indirect pathway’ leads to excessive excitatory output in the BG–SNr/GPi, either directly or indirectly through disinhibition of the STN^45^. This effect is reinforced by disinhibition of the ‘direct pathway’; the net effects from both pathways lead to overactivation of the SNr/GPi–thalamus pathway and inhibition of the cortex^45,46^. Subsequently, targeting the high firing rate in the STN using surgeries involving ibotenic acid and radionics radiofrequency lesion were first attempted, and were shown to alleviate parkinsonism^48,52,53^. These lesion surgeries were speculated to reduce the firing of STN neurons and ultimately lead to thalamus disinhibition and cortex activation. In 1995, high-frequency STN-DBS was introduced as an alternative to lesion-based surgeries^49^ and was translated to PD patients in 1995^50^. Schematic representation of altered motor circuit was adjusted from^45^. Images created with BioRender. Abbreviations: DBS, deep brain stimulation; GPe/i, globus pallidus externus/internus; IBO, ibotenic acid; MPTP, 1-methyl-4-phenyl-1,2,3,6-tetrahydropyridine; MCx, primary motor cortex; NHP, non-human primate; PD, Parkinson’s disease; SNr, substantia nigra pars reticulata; STN, subthalamic nucleus; Str, striatum; Thal, thalamus.

This translational process is depicted in Fig. 1. The development of an MPTP-treated PD NHP model—exhibiting motor symptoms (e.g., akinesia, rigidity, postural tremor, flexed posture) and pathological changes (e.g., loss of dopaminergic neurons in the substantia nigra (SN)) resembling human PD^42^—promoted the understanding of neuronal mechanisms underlying PD symptoms. Extracellular recording in this model using glass-insulated platinum-iridium microelectrodes uncovered a significant increase in tonic neuronal discharge in the GPi and STN, but a decrease in the mean tonic discharge rate in the external part of the globus pallidus (GPe) neurons^51^ (Fig. 1). The hypokinetic symptoms in PD were postulated to be the outcome of excessive tonic and phasic inhibition of thalamocortical projections due to the excitatory drive from the STN and further increased excitability of BG output—GPi/substantia nigra pars reticulata (SNr)^45^. The proposed functional model provided new insight into the theoretical basis for the surgical treatment of PD and raised the possibility that inactivation of the STN in PD would ameliorate motor deficits^45,46^. This hypothesis was verified using injection of ibotenic acid in the STN, which reduced motor disturbances (including akinesia, rigidity, and tremor) in the contralateral limbs of MPTP-treated African green monkeys^48^. Two other studies also showed that STN nucleotomy (radionics radiofrequency lesion) caused remarkable symptom reversal in MPTP-treated cynomolgus monkeys^52,53^. These studies suggested that motor improvements were obtained by functionally impairing the STN^48,52,53^. Meanwhile, high-frequency DBS—which causes reversible incapacitation of the target nucleus—was thought to exert an impact that was identical to lesion-based surgeries^49^. The effectiveness of STN-DBS was then explored in two unilaterally MPTP-treated monkeys, in whom rigidity and bradykinesia were alleviated without inducing dyskinesia, indicating the potential inclusion of STN-DBS in patient therapy^49^. In 1995, the effectiveness of STN-DBS was initially confirmed in PD patients, with noticeable improvements in akinesia and rigidity following stimulation^50^. These results closely align with findings observed in MPTP-treated monkeys^48,49,52,53^. Pollak, Benabid and colleagues translated the results of the animal models only a few months later into the first human application of STN-DBS, which was reported in 1993^54^.

From their case series larger clinical trials emerged proving the efficacy and safety of STN-DBS in PD and ultimately leading to European regulatory approval in 1998 and US Food and Drug Administration approval in 2002.

**Fig. 2:**
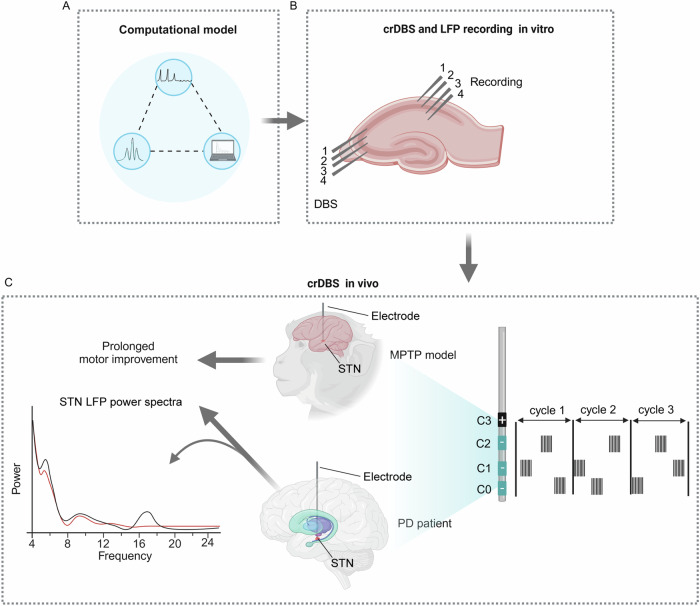
Translational process of crDBS. **A** The computational model^55–57^ and subsequent **(B)** crDBS alone with LFP recordings on rat hippocampus in vitro (adapted from^58^ with permission from the American Physical Society) indicated that crDBS caused enduring desynchronization. **C** In vivo study in which STN-crDBS showed both acute and long-lasting aftereffects on motor function in the MPTP-treated NHP model^59^. This method has also been validated in PD patients, showing that STN-crDBS in an externalized setting over three stimulation days resulted in a significant reduction in peak beta power (adapted from^60^, under the Creative Commons CC BY license), correlating with prolonged motor improvement^60^. Image created with BioRender. crDBS coordinated reset deep brain stimulation, LFP local field potential, MPTP 1-methyl-4-phenyl-1,2,3,6-tetrahydropyridine, NHP non-human primate, PD Parkinson’s disease, STN subthalamic nucleus.

Another example of translational DBS development is coordinated reset DBS (crDBS) (Fig. 2). Early computational model studies demonstrated proof-of-theory feasibility for crDBS targeting neural subpopulations^55–57^. In vitro studies showed that crDBS caused enduring desynchronization between hippocampal neuronal populations, concurrent with a widespread reduction in the amplitude of epileptiform activity^58^. Further in vivo study with an MPTP-treated NHP model demonstrated that STN-crDBS (2 h/day on five consecutive days) had both acute and long-lasting (up to 30 days) aftereffects on motor function^59^. This method has also been translated into a human study with six PD patients, which found that STN-crDBS in an externalized setting over three stimulation days resulted in a significant reduction of peak beta power (8–35 Hz) that correlated with a significant and cumulative motor improvement of 58%^60^. Unfortunately, there was no larger confirmatory clinical trial until today and STN-crDBS is still not ready for clinical application.

## Advances in neuromodulation methods

### Adaptive DBS (aDBS)

**Fig. 3:**
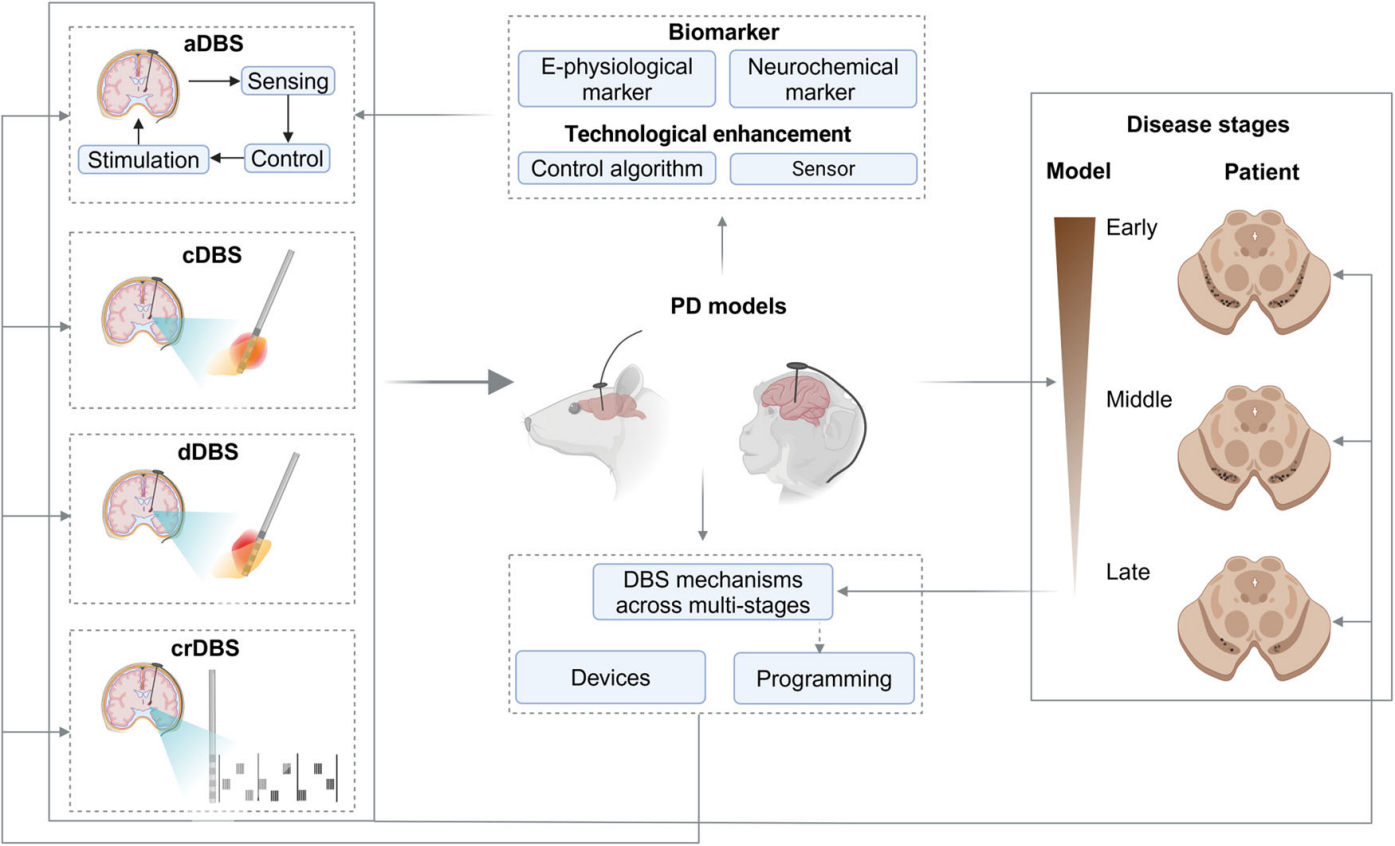
Translational approaches for enhancing DBS efficacy and safety. Several aspects can be investigated in translational animal models: (i) For aDBS, research can focus on identifying biomarkers (including both electrophysiological or chemical markers) that can be used for feedback control, enhanced sensing techniques for marker detection, and algorithms for the control unit for marker decoding and stimulation pattern design. (ii) For all DBS techniques, understanding the mechanisms behind the therapeutic effects can help improve DBS strategies, such as target selection and parameter settings. (iii) Research can explore the stability or feasibility of new stimulation devices in animal models. (iv) The effects of current well-established cDBS on different disease stages can be investigated and the optimal timing determined for achieving maximum motor and neuroprotective outcomes. (v) The feasibility of applying non-conventional DBS techniques (such as aDBS, crDBS, or dDBS) at different stages of the disease can be assessed, especially their long-term efficacy compared to cDBS. Images created with BioRender. a/c/cr/dDBS adaptive/conventional/coordinated reset/directional deep brain stimulation, E-physiological electrophysiological.

In contrast to conventional DBS (cDBS), which employs uninterrupted stimulation with constant modulation parameters, aDBS delivers personalized stimulation based on extracted feedback variables, performed in a closed-loop manner^61^. aDBS is only activated when a feedback signal threshold is reached, and aims to selectively modulate symptom-related pathological biomarkers. This technique typically consists of three modules (Fig. 3). The ‘sensing module’ first captures the known feedback variable from a specific brain region; the ‘control module’ then extracts the biomarkers and designs an optimized stimulation paradigm, and the ‘stimulation module’ delivers the stimulation^61^. In PD, the safety, tolerability, and effectiveness of aDBS have been proven, with some clinically superior advantages over cDBS. For example, the initial STN-aDBS driven by STN beta activity has shown similar^62,63^ or even better^64,65^ symptomatic benefits over cDBS in advanced PD. aDBS is also an energy-efficient approach^63–65^, and reduces stimulation-related side effects (e.g., L-dopa-induced dyskinesia, speech deterioration) in PD^63,66^. The emergence of the dual-threshold neural aDBS scheme allows for adjustable DBS intensity (increase, decrease, or maintain) based on the recorded STN beta amplitude (13–30 Hz), contributing to the maintenance of desired beta activity^67^. The first study using the dual-threshold aDBS demonstrated greater efficiency than cDBS in PD patients with tremor- and bradykinesia-dominance^67^.

#### Decoding specific feedback control markers

The successful aDBS trials in PD are largely attributed to the understanding of STN beta oscillations. The increased beta activity, ranging from 13 to 30 Hz, was initially recorded in the STN of patients through implanted DBS leads and was hypothesized to be a marker of PD motor symptoms^68,69^. Subsequent evidence supported this hypothesis, with promising findings that motor improvement (e.g., bradykinesia and/or rigidity) following either medical treatment (e.g., L-dopa) or STN-DBS was concurrent with beta power suppression in the STN^70–72^. This finding paved the way for aDBS treatment in PD, using the STN beta signal as a feedback control marker. Although some patient studies have shown the feasibility of aDBS, this technique is still not applicable for clinical use; the pathological beta oscillations in STN, for example, may not be consistent across all PD patients^61^. Beta dynamics in STN are not PD motor symptom-specific^73^ and can be affected by voluntary movements (initiation or execution)^74,75^. In the MPTP-treated PD model, aDBS reduced rigidity but not bradykinesia during a reaching task, with the interpretation that reach-related reductions in beta amplitude may disturb the timing and duration of aDBS^76^. These properties limit the long-term application of aDBS in daily life, considering the varying motor conditions. Thus, the discovery of a more specific feedback control marker is imperative.

To achieve this purpose, the identification of markers needs to extend to brain areas outside the STN. For instance, using cortical gamma power (60–90 Hz) as a feedback marker to up- or downregulate the stimulation intensity was found to reduce dyskinesia and energy consumption while maintaining therapeutic efficacy in PD^77^. Using upper limb motor activity recorded from the primary motor cortex (MCx) as the feedback variable in movement-based aDBS in three ET patients, both in the clinic and in a home environment, demonstrated the stability of tremor suppression over a longitudinal observation for 6 months^78^. Moreover, feedback markers should not be limited to spectral power but may also include a broader range of signal patterns. In PD, certain rhythmic features such as burst activities—which already show a close correlation with motor signs of PD^79,80^—may also be considered in addition to beta power.

Animal models provide a platform for marker investigation both inside and outside the STN. Bilateral STN-DBS in the 6-hydroxydopamine (6-OHDA) model, which exhibited increased MCx high beta power (20–30 Hz), found that STN stimulation disrupted the pathological high beta oscillations, thus restoring motor control^81^. In line with this study, PD patients exhibited increased high beta power (21–35 Hz, compared to ET patients) in the MCx. Beta-burst analyses further revealed that burst durations of the MCx beta band were positively correlated with the aggregate contralateral limb UPDRS score^82^. Another study reported that high-frequency STN-DBS effectively suppressed MCx beta-burst amplitude, with concurrent motor improvement^83^. These studies strongly indicate that MCx beta activities, including spectral power and burst patterns, may also be markers of PD motor symptoms or predictors of DBS response. Increasing patient and model evidence suggests that other signal signs in PD are also linked to motor deficits, such as phase-amplitude couplings^84–86^, aperiodic components^87,88^, and waveform shape^82,89^.

Of note, neurochemical markers are also under investigation in basic research, in addition to neuronal markers (Fig. 3). A wirelessly controlled stimulation device (Mayo Investigational Neuromodulation Control System, MINCS) synchronized with a wirelessly controlled neurochemical recording device (Wireless Instantaneous Neurochemical Concentration Sensing System, WINCS) has been reported^90^. Results demonstrated that the sensors of these devices can measure changes in striatum dopamine in the rat brain using fast-scan cyclic voltammetry in vivo and transfer the chemical signal in an artifact-free manner^90^. In the 6-OHDA rat model, STN-DBS induced a measurable glutamate response in the globus pallidus (GP), suggesting that measuring GP glutamate concentrations may provide a feedback-control signal for aDBS in PD^91^. Thus, in these feedback control stimulation pipelines, neurochemical recordings serve as the feedback variables used to adjust stimulation parameters. This is potentially important for aDBS treatment in PD patients who do not express pathological beta activity.

Furthermore, animal studies can help to identify specific markers corresponding to different stages of the disease (Fig. 3), which may not be available in human studies with limited sample sizes and unavailable histological information. Given the current aDBS strategy targeting only advanced PD with extreme nigrostriatal depletion and severe motor symptoms, less is known about the feasibility of aDBS for PD in earlier stages. It is necessary to uncover the intrinsic connections between pathological markers and motor symptoms and further test the feasibility of using these markers on established aDBS translational platforms, such as aDBS platforms in the 6-OHDA rat model^92^ or MPTP-treated NHP models^76,93,94^.

#### Improved aDBS algorithms

Another approach to improving aDBS is enhancing the control algorithms (Fig. 3). Current studies regarding aDBS control algorithms are mainly based on computational models. These studies are essential because they provide a theoretical basis for the application of algorithms^95,96^. However, before applying them to patients, it is necessary to test their efficacy and safety in translational animal models. For example, comparing the efficacy of different control algorithms helps to identify the optimal algorithm for aDBS^92^. Moreover, new control algorithms with enhanced biomarker decoding and DBS parameter organization capabilities need to be designed towards different feedback control markers (Fig. 3).

### dDBS

Conventional DBS utilizing ring-shaped electrodes generates omnidirectional stimulation through a spherical electric field, leading to the spread of the electric field and potential stimulation-induced side effects due to spillover into adjacent structures^5,97^. In contrast, emerging technological innovations such as dDBS offer the ability to control the distribution of the electric field exclusively toward the area of interest (e.g., the motor sector of the STN in PD), while avoiding adjacent structures such as the pyramidal tract (Fig. 3)^5^. Directional DBS has demonstrated several advantages over cDBS, including a wider therapeutic window, greater reduction in daily medication doses, and improvements in health-related quality of life^98,99^. Clinical controlled trials of this technology are currently ongoing, primarily focusing on STN-DBS in PD patients. This method has been successfully transferred to healthy rodents^100^, where orientation-selective STN-DBS targeting the mediolateral STN led to the strongest activation of the sensorimotor and BG–thalamus–cortex networks. So far, this technology has not been translated into animal disease models.

### crDBS

The implementation of electrical crDBS involves delivering high-frequency pulse trains through distinct contacts (Fig. 2C). This weak stimulus does not block the neuronal firing but resets the phase of the targeted neurons^56,58,59^. As the pulse trains are delivered through different lead contacts in a cyclical manner at different times (Fig. 2C), the neuron population is divided into phase-shifted subpopulations, ultimately leading to network desynchronization^56,58,59^. The advantages of this technique over cDBS include prolonging the stimulation effects^58,60,101^ and the ability to deliver stimulation in an on-demand manner with lower pulse amplitude and frequency, resulting in fewer side effects and lower energy consumption^56,101^. This preliminary evidence demonstrates the effectiveness of crDBS for clinical use and targeting PD patients with abnormal synchronized activities (e.g., beta oscillation) in DBS targets. However, its long-term efficacy and safety need to be examined through prospective, large-scale clinical trials.

### Temporally optimized patterned stimulation (TOPS)

Using an intraoperative stimulation protocol, temporally non-regular patterns of STN-DBS were found to be as effective as cDBS in modulating motor and beta activity^102^ or even superior in some cases^103^. A proof-of-concept study utilizing a BG computational model coupled with a genetic algorithm (GA) successfully optimized stimulation patterns^104^. This study demonstrated, through direct translational application from 6-OHDA-lesioned rats to PD patients, that optimized temporal DBS patterns achieved symptom relief at lower stimulation frequency. The feasibility of TOPS was further validated in a randomized, prospective, multi-center study^105^. In general, TOPS consistently reduced energy consumption compared to cDBS^102–105^, aligning with the concept of personalized treatment and suggesting that patient-specific models could be used for designing stimulation patterns^104^.

### The DBS system

#### DBS parameters

Clinically, DBS programming is a crucial step in maximizing the benefits and minimizing both adverse effects and the energy consumption of the implantable pulse generator (IPG) battery^106^. However, programming follows a demanding trial-and-error approach^107^ and is executed through the individual titration of the dose of electrical stimulation^108^. It has been reported that for STN-DBS in PD, a ceiling beneficial effect is obtained in the high-frequency range from 130 to 185 Hz, with a pulse width set at 60 μs^106^. Currently, reverse translation studies on animal models predominantly use patient-comparable stimulation settings, typically involving a combination of frequencies over 130 Hz, a pulse width of 60 μs, and either fixed/side effects-dependent current or constant voltage control^109^. These studies are mainly aimed at investigating the underlying mechanisms of DBS instead of looking for optimized stimulation parameters. Indeed, parameter settings for patients are much more complex and should comprehensively consider therapeutic benefits, side effects, pharmacological management, and adverse events such as speech, gait, balance, neuropsychiatric events, etc.^110^. In contrast, animal studies mainly focus on assessing therapeutic effects, often with a primary emphasis on side effects such as dyskinesia^111^. This likely accounts for the poor translation of DBS parameters from model to patients.

**Fig. 4:**
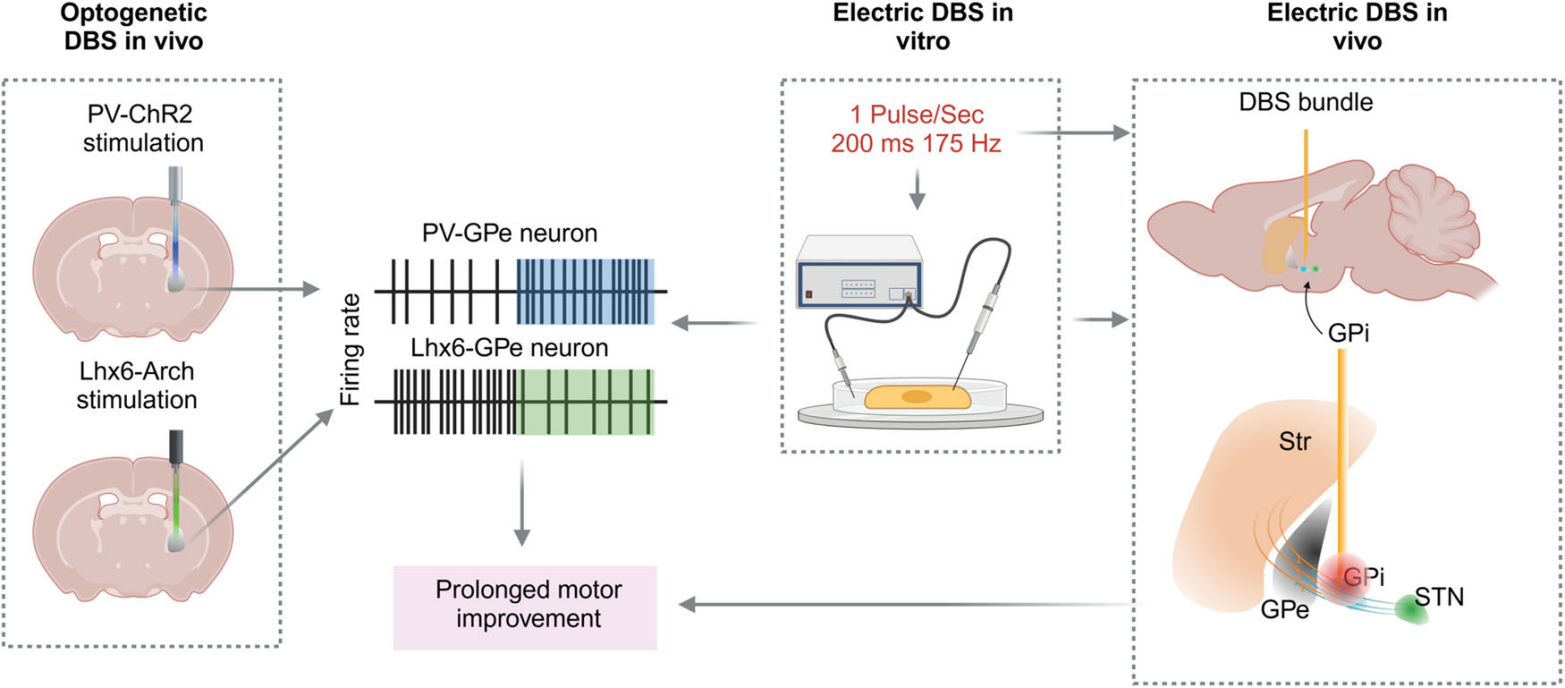
An example of cell type-specific DBS. In 6-OHDA lesioned mice, selectively activating PV-GPe neurons (increased firing rate) or inhibiting Lhx6-GPe neurons (decreased firing rate) led to prolonged motor improvement^114^. This cell type-specific modulation was reproduced using electrical stimulation in vitro on PD mice slices, using parameters of one DBS pulse/sec, a pulse width of 200 ms, and an intraburst frequency of 175 Hz^9^. Using the same DBS parameters, electrical stimulation of a spot near the EPN, where the STN and D1-SPNs efferents could be coactivated, led to long-lasting therapeutic benefits^9^, as seen in the optogenetic study^114^. Images created with BioRender. 6-OHDA 6-hydroxydopamine, DBS deep brain stimulation, EPN entopeduncular nucleus, GPe/i globus pallidus externus/internus, PD Parkinson’s disease, PV parvalbumin, SPNs spiny projection neurons, STN subthalamic nucleus, Str striatum.

Clinically, determining optimal, patient/symptom-specific stimulation parameters remains a challenge and there is still a lack of consensus on parameter settings. Achieving the best stimulation parameters requires a highly trained clinician and can take 3–6 months to obtain optimal results^110^. The intrinsic logic behind this process is to select parameters that are responsible for achieving the best balance between benefits and risks. In this context, a symptom-specific concept arises that aims to target only symptom-related neuron elements. In translational studies, the symptomatic benefits of DBS are dependent on the frequency-specific activation of specific cell types^112,113^ and the optimal composite metric parameters setting for DBS in both in vitro and in vivo studies^9,114^ (Fig. 4; a detailed description can be found in Cell type-specific electrical stimulation). Consistent with this concept, low-frequency DBS using the TOPS pattern^105^ and the dual-frequency paradigm known as interleave-interlink (IL-IL)^115,116^ have been reported as potential approaches for personalized therapy. The latter strategy utilizes low-frequency stimulation to target axial symptoms (e.g., gait and balance) while preserving control over appendicular symptoms by high-frequency stimulation^115,116^. However, these parameter settings have not yet been assessed in PD models. Finally, considering that automated data-driven algorithms (such as StimFit) can predict DBS parameter settings leading to motor improvement in PD patient studies^107^—reverse translational studies can apply this method to basic research to assess its applicability across different DBS strategies.

#### Device improvement

The development and characterization of novel technologies are needed for preclinical testing on animals. For example, the functionality of a new lead with 40 contacts in five columns was tested in an MPTP-treated NHP model, and demonstrated enhancements in the spatial direction of stimulation and the spatial precision of LFP recordings^117^. Another translational study tested the burr hole device and lead design in ovine models, focusing specifically on lead stability^118^.

In general, animal model studies contribute to the evaluation of neuromodulation methods at various aspects, including hardware, biomarker, control algorithms, and DBS parameters (Fig. 3). Some DBS patterns have been directly assessed in human subjects, however, these evaluations are often limited to short observation periods. A comprehensive assessment of the efficacy, safety, and tolerability of DBS methods for long-term application or across various stimulation windows (e.g., early stimulation initiation) is essential in preclinical models. Insights gained from animal studies on the mechanisms of stimulation can further optimize DBS therapy (Fig. 3).

## Targeting symptom-specific neuromodulation

The efficacy of STN-DBS on the MPTP-treated NHP model laid the groundwork for ‘model to patient’ translation. This representative case demonstrated the importance of the evidence-based translational process regarding new target discovery (Fig. 1). The translating process was based on an understanding of BG functions^7^, which are implicated in many important, segregated, parallel thalamocortical circuits (e.g., motor circuits)^43,47^. The successful translation from the PD NHP model to patients with STN-DBS led to the discovery of targets for DBS that could improve akinesia and gait impairment in PD^7^. Evidence showed that unilateral low-frequency (2.5, 5.0, and 10 Hz) PPN-DBS effectively reversed akinesia in the MPTP-treated NHP model^119^ and suggested that PPN-DBS may be a feasible target for advanced PD with akinetic disorders^119^. However, over decades of investigation, the exact therapeutic efficacy of PPN-DBS in PD patients has not been confirmed. Limited patient studies have shown varied and even controversial treatment outcomes across centers^120–124^.

The translational study of PPN-DBS highlights the limitations of directly transferring findings from NHP models to patients, especially concerning selection of the DBS target. The varied methodologies across treatment centers may be attributed to clinically unclear fundamental aspects of PPN-DBS application, such as specific markers of treatment response, optimized DBS spots, and DBS parameters^125^. In this regard, animal studies are crucial for understanding the modulation mechanisms that target cell types and specific modulation of oscillatory activities at the mesoscale. These studies provide opportunities to explore symptom-specific neuromodulatory mechanisms at the cerebral circuit level, informing the directions of future neuromodulation research^126^ and potentially indicating modulation strategies, including target selection and stimulation parameter adjustment.

### Symptom-specific neural oscillations

Determining optimal modulation regions, known as ‘sweet spots’, is a vital aspect of achieving the best stimulation outcome. Several retrospective studies have highlighted the pivotal role of evidence-based, sweet-spot definition, focusing on local oscillatory activities. For instance, in PD the optimal stimulation site was mostly defined as the dorsolateral part of the STN^127,128^, located within the sensorimotor functional sector^129^. This definition was supported by an electrophysiological study. After projecting the beta power (13–35 Hz) into a common stereotactic space following electrode localization, the area with the maximal expression of beta power was located in the posterior and dorsal lead positions^130^. Similarly, sweet-spot mapping was also applied in cervical dystonia patients, which showed that the peak power of dystonia symptom-related theta oscillations (4–12 Hz) was located in the posterolateral GPi^131^. The proximity between the active contact and this peak area, which correlated with dystonia, suggested that this area could be used as the sweet spot of GPi-DBS in dystonia^131^. Moreover, LFP recordings from the PPN have reported alpha oscillations (6–12 Hz) in the caudal PPN and beta oscillations (12–30 Hz) in the rostral PPN^132,133^. This alpha power was linked to gait speed and freezing^132^. These studies also suggested that the optimal subregion of PPN-DBS was located in the caudal PPN rather than the rostral part^132^. These insights gained from patient studies regarding the sweet spot may potentially help with intraoperative electrode placement or programming.

Translating methods from human to animal models may offer insights into understanding the mechanisms of sweet-spot stimulation. In a study involving healthy rats, the maximal activation of the sensorimotor and BG–thalamus–cortex networks occurred when the electric field was located in the STN pointing laterally in the mediolateral direction^100^. However, no studies in PD models have explored modulation of specific subregions within the STN. One obstacle is the small size of rodent brain structures, which limits the spatial mapping of LFP or imaging signals. The introduction of a large-scale, electrophysiological recording technique called Neuropixels^134,135^ may help to address this issue.

### Symptom-specific cell populations

The concept of cell type-specific modulation is derived from optogenetic studies in animal models, demonstrating significant symptomatic benefits^112–114,136,137^. For example, in 6-OHDA unilateral lesion rats, high-frequency optogenetic stimulation of the STN—with the expression of a glutamatergic neuronal element-specific viral vector (Chronos)—showed amelioration of motor deficits, particularly characterized in forelimb stepping^113^. Similar symptomatic effects, characterized by mean velocity in an open field test, were also found in parkinsonian vesicular glutamate transporter 2 (VGlut2)-Cre mice with Cre-dependent channelrhodopsin-2, followed by pulsatile, 50 Hz, blue light stimulation^112^. Notably, the former study^113^ also found suppressed high beta power (20–30 Hz) in the STN and SNr under optogenetic DBS, further indicating that the cell-specific optogenetic activation of local STN neurons was sufficient to alter neural firing patterns^113^, similar to the effect observed with STN-DBS^71,138^.

Previous studies have shown that intraoperative microelectrode recording of single-cell neuronal activity could identify the border of the STN^139^ and may be feasible for the determination of the motor target for STN-DBS^140^. The latter study reported a 41% and 33.3% bursting pattern in the dorsal and central STN, respectively, but only 19% of this pattern in the ventral part^140^. This raised great interest in elucidating the cell populations responsible for this firing pattern in the dorsal STN. Indeed, identifying the specific cell population behind the symptom-related firing rhythms is not only beneficial for designing the best placement of DBS electrodes to maximize therapeutic effects, but also to reduce unwanted behavioral and cognitive side effects^141^. Using healthy mice, two functional types of glutamatergic STN neurons (parvalbumin (PV)+/-) have been reported in the STN^142^: the glutamatergic PV+ neurons are located in the dorsolateral and middle STN, contribute to burst firing, and are segregated topographically, while PV- neurons are more distributed in the ventral STN and express tonic firing. Given that this topographic burst pattern in healthy mice was comparable to human findings in PD patients^140,143^, this study raised the question of whether this bursting mode is only a firing nature of PV+ neurons in medial-dorsal STN or whether it is PD pathology-relevant after progressive dopamine depletion. Subsequent studies on PD models should clarify the bursting patterns of PV+ neurons across PD and control states and their relationship with motor symptoms.

Additionally, it has been hypothesized that the non-consistent clinical outcomes following PPN-DBS may be because of non-specific electrical stimulation of PPN neuron populations^137^. Indeed, the PPN is heterogeneously complex and consists of glutamatergic, cholinergic, and GABAergic cell populations^144^. A recent study showed that cell-specific activation of glutamatergic neurons in the caudal PPN reverted akinesia and bradykinesia mediated by the dopamine D1 antagonist SCH23390 and the D2 antagonist haloperidol, while activation of caudal GABAergic PPN neurons did not produce a bradykinetic phenotype^137^. This study suggested that the concurrent stimulation of glutamatergic and GABAergic neurons probably leads to varied outcomes in clinical trials^137^. Therefore, directing interventions towards the glutamatergic neuronal populations in the caudal PPN could potentially yield optimal DBS outcomes for locomotor function.

### Cell type-specific electrical stimulation

It has been suggested that translating optogenetic findings to human neuromodulation requires cell type-specific artificial expression of chemical or optical actuators^137^. The effectiveness of optogenetic DBS has been shown to be frequency dependent^145^, with the best motor relief and beta suppression found under 130 Hz stimulation, mimicking the effects of electrical stimulation using the same frequency. In another study on parkinsonian mice, significant motor improvement was only observed with 50 Hz optical DBS rather than continuous stimulation. This also indicated that locomotion improvement was frequency dependent^112^. These studies led to the hypothesis that activation of a specific cell type is frequency sensitive. Striking findings of cell type-specific modulation by DBS have been reported, along with persistent motor improvement in 6-OHDA lesioned mice^9^ (Fig. 4). This work was based on an optogenetic study using the same mouse model, which showed that selectively activating PV-GPe neurons or inhibiting Lhx6-GPe neurons could restore motor function, and that persistent behavioral rescue depended on the ratio of PV and Lhx6 neurons in the GPe^114^. Delivering short DBS bursts once per second, with a duration of 200 ms and an intraburst frequency of 175 Hz, showed inhibition of Lhx6 neurons but excitation of PV+ neurons in GPe slices of the mouse model^9^. Subsequent DBS in vivo with lead location around the entopeduncular nucleus, where STN efferents and D1-spiny projection neurons could be coactivated, led to long-lasting therapeutic benefits^9^, as seen in the optogenetic study^114^. These studies demonstrated that the neuron population specificity of electrical stimulation can be refined^9^. The effects of electrical stimulation, akin to optogenetic interventions, depend on the adjustment of stimulation parameters and the selection of stimulation spots.

Taken together, the identification of symptom-related cell dynamics, adjustment of modulation parameters, and selection of stimulation spots are crucial steps for cell type-specific stimulation. Such fundamental knowledge is not available in patients, only in animal and computational models. Cell type-specific optogenetic studies have also inspired new target discoveries, such as targeting VGlut2-positive neurons in the cuneiform nucleus^136^, and activation of the A13 region (medial zona incerta)^146^.

## Targeting disease-modifying effects

### Symptomatic benefits of earlier STN-DBS in PD

In PD, the stage following disease onset is referred to as the honeymoon period, during which medication therapy effectively improves motor symptoms^10^. As the disease progresses into the intermediate period, L-dopa-induced motor fluctuations and dyskinesia become evident^10^. Performing DBS surgery soon after the diagnosis of PD has been associated with symptomatic benefits and improved quality of life^13,147–150^. In an older retrospective analysis, three out of 41 patients with a disease duration of less than 10 years were able to maintain their professional activity after DBS implantation^150^. Subsequently, a randomized controlled trial from the EARLYSTIM Study Group implemented bilateral STN-DBS in patients with mild to moderate motor symptoms and a mean disease duration of 6.8 years. Motor signs decreased by 69% in the DBS-treated group (*n* = 10), compared to a 29% increase in patients receiving best medical treatment (BMT, *n* = 10)^147^. In 2013, the EARLYSTIM Study Group reported an investigator-initiated, randomized, multicenter, parallel-group study involving 251 patients in the early onset of motor complications stage^13^. Patients were treated with either neurostimulation + BMT or BMT only. The study revealed that neurostimulation was superior to BMT in terms of motor function outcomes. Overall, the clinical evidence suggests that early STN-DBS may have a long-lasting symptomatic benefits and a potential disease-modifying effect.

To better understand the disease-modifying effects of early DBS, a progressive adeno-associated virus (AAV) 1/2-driven human-mutated A53T alpha-synuclein (aSyn)-overexpressing PD rat model (AAV1/2-A53T-aSyn, 2.5 × 10^12^ gp/mol) was utilized^151–153^. In this model, motor improvement (evaluated by the single pellet reaching task) was maintained even after turning off the stimulator for 24 h following 3 weeks of unilateral STN-DBS, indicating the disease-modifying effects in PD^111^. Similar effects were also observed in the intrastriatal 6-OHDA lesioned PD model, where a significantly lower number of amphetamine-induced rotations was found in the off-state stimulated group compared to the unstimulated group^154^. These rodent studies provide strong evidence to support the symptomatic benefits of earlier DBS strategies and raise the possibility that early stimulation could exert long-lasting motor protection for PD patients over the course of the disease.

### Neuroprotective effects of STN-DBS in PD

To date, no clinical evidence demonstrates neuroprotection following STN-DBS in earlier PD stages. One reason for this is the lack of postmortem studies, which include the evaluation of PD pathology such as SN depigmentation to investigate the neuroprotective effects of STN-DBS on PD^155^. In contrast, the quantification of neurodegeneration in PD with or without long-term DBS is available in animal studies. Increasing preclinical evidence suggests that chronic STN-DBS protects dopaminergic neurons in the SN. In an early foundation study using a rat model of PD with intrastriatal 6-OHDA injection, unilateral 2-week STN-DBS initiated immediately after the 6-OHDA injection demonstrated a remarkable neuroprotective effect. The stimulated PD group exhibited a significant >15% reduction in dopaminergic neurodegeneration relative to the intact side, while the non-stimulated group experienced severe degeneration at around 56% relative to the intact side. This compelling result suggested that STN-DBS holds the potential to effectively protect dopaminergic neurons in the substantia nigra pars compacta (SNc)^154^. Subsequent studies using the same model with intrastriatal 6-OHDA injection showed similar neuroprotective effects of STN-DBS^156–161^. However, in a 6-OHDA rat model with medial forebrain bundle (MFB) injection, long-term unilateral STN-DBS did not result in neuroprotective effects following 3 or 6 weeks of DBS, or 3 weeks of DBS followed by a 3-week washout period^162^. In contrast to MFB lesions with 6-OHDA, the intrastriatal lesioned model is thought to be a progressive PD rodent model^163^. The progressive PD with surviving dopaminergic neurons and ongoing degeneration is a key factor for a neuroprotective effect. Indeed, using a progressive AAV1/2-A53T-aSyn (2.5 × 10^12^ gp/mol) PD rat model, it was found that 3 weeks of STN-DBS protected dopaminergic neurons by approximately 29% in the SN^111^. In an MPTP-treated monkey (which generated a subacute dopaminergic cell loss of ~50%), high-frequency STN-DBS led to a neuroprotective effect, preserving around 20% of SNc dopaminergic cells after stimulation^164^. These studies suggest that STN-DBS improves the motor symptoms of PD—but also has the potential to slow down disease progression^164^. While current research suggests that STN-DBS offers a neuroprotective effect in PD, definitive evidence remains elusive due to limitations in histological assessments of PD patients. Neuroimaging surrogate markers have been identified, reflecting nigrostriatal degeneration. For example, [^18^F]Fluorodopa uptake measurements has been shown to correlate with dopaminergic cell counts in postmortem SN tissue from a small group of PD patients^165^. Longitudinal studies with larger patient cohorts would provide a more comprehensive understanding of how this marker evolves and correlates with progression of neurodegeneration. So far, it remains unknown whether this surrogate accurately reflects the number of dopaminergic neurons in the SN and terminals in the striatum of alive patients^166^. Therefore, a reliable and direct biomarker to monitor the neuroprotection responding to DBS intervention still needs to be identified.

### Identification of the best timing for early DBS

A substantial body of preclinical studies has demonstrated the disease-modifying effects of early STN-DBS in PD. However, translating these findings to clinical treatment presents challenges, particularly in determining the optimal timing for maximizing these plastic benefits. A postmortem study comparing PD patients with bilateral STN-DBS to a non-DBS group found that STN-DBS subjects tended to have higher aSyn density scores, but there was no differential loss of SN pigmented neurons^155^. This lack of neuroprotective effect might be attributed to the advanced disease duration (averaging 18.9 years) in the STN-stimulated subjects^155^. In advanced PD, where the nigral cell death rate reaches approximately 85%, patients in the earliest symptomatic stages have much lower nigral cell loss rates (~50%)^167^.

Current considerations for DBS interventions primarily depend on disease severity, treatment-related complications, and disability, rather than following a time-dependent disease course^168^. The early stage of human PD can be defined as the onset of the so-called intermediate phase, situated between the honeymoon phase and the late phase, during which L-dopa-induced dyskinesia has manifested^10^. Motor fluctuations were reported to occur in up to 80% of PD patients after 5 to 10 years of L-dopa treatment^169^. This was likely due to the substantial decline (ranging from 50–90%) in dopaminergic fiber density in the dorsal striatum within 4 years after the initial diagnosis^170^ and repeated L-dopa administration. Therefore, PD patients who have already experienced the onset of dyskinesia likely exhibit severe fiber denervation in the striatum, possibly exceeding 80% fiber loss. It has been suggested that including PD patients with >4 years of disease duration may pose challenges for ‘neuroprotective’ therapies^170^. The established nigrostriatal denervation may lead to a poor neuroprotective benefit of STN-DBS^167^. Of course, the exact state of nigrostriatal depletion cannot be quantified precisely and directly in patients. In animal models, characterizing disease progression^152^ and applying DBS at different disease stages would provide insights into the optimal time window for achieving maximum disease-modifying effects.

### Understanding the disease-modifying effects of DBS

From a translational perspective, understanding the neuroprotective mechanisms in preclinical PD models can inform an optimized strategy for the disease-modifying potential of STN-DBS in clinical treatment^156^. Two proposed mechanisms based on current findings are outlined below.

#### Reduced excitotoxicity in the SNc

The ‘excitotoxicity’ hypothesis is rooted in the anatomical relationship between the SNc and STN, along with the pathological activation of the STN under PD conditions^171^. Speculation suggests that the overactive STN may increase glutamate excitotoxicity in the SNc, leading to further dopaminergic neuronal loss^164,171^. STN-DBS is hypothesized to reduce glutamatergic input to the SNc, resulting in neuroprotection^164^. However, direct evidence supporting this mechanism is currently lacking.

#### Activation of BDNF-TrkB signaling

The brain-derived neurotrophic factor-tropomyosin receptor kinase B (BDNF-TrkB) hypothesis is supported in the literature. For example, Spieles-Engemann et al. demonstrated that 2 weeks of STN-DBS led to bilateral upregulation of the BDNF protein in the nigrostriatal system (SN and striatum) and the MCx^161^. Additionally, increased BDNF mRNA was observed in both the GPi and the SN in the intrastriatal 6-OHDA lesioned rat model^161^. In the same model, increased BDNF-TrkB signaling contributed to the neuroprotective effect of STN-DBS^156^. This was evidenced by the effective activation of the BDNF-TrkB signal pathway by STN-DBS, exhibited as the phosphorylation of Akt and ribosomal protein S6 in SN neurons^156^. Further bolstering this mechanism, administration of the TrkB blockade ANA-12 led to the abolition of the STN-DBS-mediated rescue of SN neurons^156^.

Clarifying the mechanisms underlying disease-modifying effects can provide valuable insights for optimizing DBS strategies—including DBS modes, timing, and parameter settings—and informing long-term outcomes. Investigations into the mechanisms of neuroprotective effects of DBS have been conducted in toxic-based models such as the 6-OHDA rat model or the MPTP-treated NHP model. Exploring these mechanisms in vector-based models, which are more relevant to PD pathology^151,153^ and have also demonstrated neuroprotective outcomes with chronic STN-DBS^111^, would be of significant interest.

## Network modulation

### Network oscillatory synchronization

Current insights into DBS action in neural circuits at multiple scales (micro, meso, and macro) have been summarized in a recent review^73^. Understanding how DBS action can interfere with neural activity in distant brain regions is crucial, involving the investigation of between-region synchronization and whole-brain coupling patterns at the macroscale^73^. PD animal models enable simultaneous recordings from multiple brain regions. The key electrophysiological oscillatory features in PD patients, such as STN beta power or burst properties, have been reproduced in rodent models^172–175^ and the MPTP-treated NHP model^145,176^ and have have shown correlation with motor phenotypes^145,175^. The findings regarding cortical beta oscillations or STN–MCx beta coupling at the macroscale in PD models^174,177,178^ also demonstrated similarity to patients^179–181^. The patient-comparable network oscillatory patterns allow the investigation of the effects of DBS on oscillations in remote brain regions or the coupling between regions.

In the 6-OHDA rat model, the recorded stochastic antidromic spikes from the STN showed direct modulation of neuron firing probability in the MCx and further disrupted the dominant high beta (20–30 Hz) oscillations, indicating that STN-DBS could directly influence the MCx in PD^81^. This finding also led to the investigation of antidromic effects of STN-DBS in an NHP PD model study, which had the head size and lead dimensions more appropriately scaled to humans^182^. Consistent with the former study^81^, antidromic activation of the primary MCx was also observed, suggesting activation of the hyperdirect pathway during STN-DBS^182^. These findings across PD models supported the hypothesis that antidromic activation of the MCx via the hyperdirect pathway is a key DBS mechanism underlying therapeutic efficacy^183^. Indeed, a functional magnetic resonance imaging study in PD patients showed that STN-DBS modulated the primary MCx circuit in a time-dependent manner, and this modulation was related to reduced bradykinesia^184^. STN-DBS has been found to normalize the cortical beta burst amplitude, and this effect is related to motor improvement^83^. These studies emphasize how fundamental work on animal models contributes to further clinical studies regarding the distant modulation of DBS.

### Network metabolism

The impact of STN-DBS on the cerebral network of PD has been explored through ^18^F-fluorodeoxyglucose-positron emission tomography (FDG-PET) studies. PET imaging provides valuable insights into the functional conditions of the BG–thalamus–cortex loop in PD. FDG-PET analysis pipelines enable the examination of metabolic activities in single brain regions using the standard uptake value or network architecture properties. It has consistently been demonstrated that PD exhibits an abnormal spatial covariance pattern known as the PD-related pattern (PDRP). This pattern is characterized by increased metabolism in the pallidum, thalamus, pons, cerebellum, and sensorimotor cortex, along with reduced metabolic activities in the lateral pre-MCx and parieto-occipital association regions^185^. The reduced PDRP activity and clinical improvement after STN-DBS suggest that modulation of pathological cerebral networks is a critical feature of the treatment response in PD^185^. A similar pattern has been identified in an MPTP-treated NHP model (adult macaque monkeys), and the spatial topography was reproducible and consistent with homologous findings in human PD^186^. However, this specific pattern has not been reported in PD rodent models.

However, results are inconsistent when examining local metabolism across cerebral networks, especially when linking motor improvement after STN-DBS to regional FDG-PET signal changes. For example, the improvement in rigidity after STN-DBS was found to be related to decreased regional cerebral blood flow (rCBF) in the supplementary MCx, and the improvement in bradykinesia was related to increased rCBF in the thalamus^187^. Another study showed that deactivation of both the supplementary motor area and the thalamus were correlated with motor improvements after STN-DBS^188^. Variable results may be attributed to dynamic metabolic changes over different disease conditions, such as varying levels of neurodegeneration in the SN or potential participation of compensatory processes.

Therefore, it would be meaningful to clarify whether the metabolic changes are disease-state dependent. Investigating the PDRP pattern in NHP models and regional metabolic changes in NHP and rodent models at a temporal scale may help to elucidate what constitutes a symptomatic or degenerative marker across different stages of the disease. Further observation of metabolic changes following DBS would help to validate markers that could be utilized to optimize DBS and help predict the treatment response of DBS.

## Inherent limitations

Animal models provide opportunities for evaluating methodologies in the preclinical stage and for studying aspects of the disease they model for that are not accessible in human research. However, caution must be exercised when interpreting or translating findings from these models due to the inherent limitations associated with inter-species and model differences.

### Brain anatomy

In humans, the complexity of the brain exhibits significant anatomical divergence from that of rodents^189^. For example, the scale of cortical regions, including both gray and white matter, is much smaller in mice compared to humans^189^. Studies of cortical circuit configuration indicate a concomitant 1000-fold expansion in neuronal network size and a 2.5-fold increase in inhibitory interneurons from mouse to human^190^. In the human brain, the caudate and putamen are distinct structures separated by the internal capsule, whereas in rodents, these two structures are fused. In contrast, NHPs display a more similar anatomy when compared to humans^191^. The MPTP-treated NHP model, which features a motor circuit comparable to that of humans, has significantly contributed to our understanding of the circuit mechanisms underlying PD and the evaluation of STN-DBS therapy (see Historical translation). Some brain regions in rodents exhibit greater structural divergence but retain functional similarities as humans. For instance, the rodent homologue of the human GPi—EPN—has been utilized in PD and dystonia studies involving DBS interventions^162,192–194^.

### Locomotor system

Behavioral assessments in rodent models do not fully capture the spectrum of motor symptoms associated with PD. For instance, rodent models typically do not exhibit tremors, a hallmark symptom in human PD. Gait analysis reveals significant differences in mechanics: humans, as bipeds with an upright posture, demonstrate distinct gait patterns compared to quadrupedal rodents. The introduction of machine learning pipelines for feature extraction in rodent gait analysis may standardize translational research from rodents to human PD^195^. This approach may clarify the intrinsic mechanisms behind common gait patterns across species. Indeed, it has been suggested that, while PD motor deficits manifest differently in rodent models and patients, the underlying neuroanatomical components governing motor control may be similar^196^.

### Pathology

Currently, none of the existing animal models can fully replicate the complete signs of PD pathology. The vector-based or rotenone rodent models exhibit human-mimicking pathologies, such as α-synuclein and Lewy body-like pathology, while the 6-OHDA rodent model does not^197,198^. In contrast, the 6-OHDA model, which induces rapid and severe loss of dopaminergic neurons in the SN, simulates advanced PD and demonstrates significant circuit dysfunction, including pronounced beta propagation in the motor circuit^177^. The high-risk factor of PD—age—poses challenges in accurately modeling the complexities of the disease’s pathology due to the inherently shorter lifespan of rodents and their faster rate of aging compared to humans. Age-dependent interactions with PD pathology can still be assessed by prolonging observation times or using aged animals. For instance, extending the observation window to 16–17 months in hm^2^α-SYN-39 mice revealed an age-dependent loss of dopaminergic neurons, along with an increase in CD8 + T cells and microglia in SN^199^.

Although NHP models exhibit greater similarity to human PD, NHP studies are less common, and sample sizes are typically limited due to ethical considerations. Conversely, rodent models remain the predominant choice for in vivo translational research. It should be noted that the selection of animal models primarily relies on the particular focus of the study design or the scientific objectives. Studies on a specific model reflect only one aspect of the disease, and therefore may not capture its full complexity. To avoid hindering translational efforts, it is crucial to understand both the equivalent and divergent characteristics across species or models, as this knowledge will facilitate more rational data extrapolation from rodents to humans^189^.

## Reverse translation of methodology

### Wireless stimulation in rodents

**Fig. 5:**
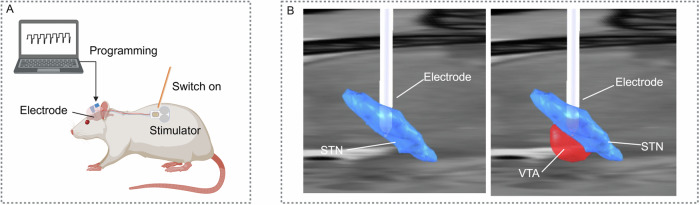
Reverse translation of methodologies from human to rodent. **A** A novel wireless stimulation system, which can be programmed through a computational interface and activated with a magnet. The electrode part is secured using dental cement, while the extension and stimulator are implanted subcutaneously. **B** Electrode modeling of a rodent DBS electrode. The lead placement is validated in three-dimensional space. DBS current (μA)-based VTA calculation showed overlapping between VTA and STN. Images created with BioRender. DBS deep brain stimulation, STN subthalamic nucleus, VTA volume of tissue activated.

The current DBS system used clinically consists of three main components: a programmable battery-powered pacemaker, an insulated extension, and leads placed inside the brain targets through burr holes. NHP studies use the same IPG as humans and a scaled-down DBS lead, which has shown closed reproduction in human settings^200^. However, traditional DBS systems for experimental rodent studies do not fully mimic those for patients. A cable-bound external stimulator connects the DBS electrode implanted in the rodent’s brain. While this configuration is widely employed, it comes with certain limitations, particularly in terms of flexibility during behavioral tests and for long-term stimulation purposes^109^. Additionally, the fixed cable connection hinders the feasibility of certain advanced imaging techniques, such as molecular imaging with FDG-PET during DBS. To address these issues, wireless microstimulator and stimulation systems have been developed^195,201,202^. These systems are more analogous to the human IPG and allows experimental DBS studies in rodents that are more comparable to human settings (Fig. 5A).

### Electrode modeling

A pipeline integrating DBS electrode localizations, computational modeling, and MRI-based neuroimaging methods has been established, enabling the analysis of DBS network modulation effects^73,203^. Recently, this pipeline for DBS electrode localizations has been reverse-translated from humans to rats^204^. In contrast to traditional nissl staining, CT scan-based electrode localization visualizes the lead position in a three-dimensional space, allowing the calculation of subject-specific volumes of activation (VTA) and pathway activation (Fig. 5B)^204^. In particular, VTA calculations and fiber activation modeling can characterize the impact of DBS on the target area^204^. Future application of this pipeline to animal model studies has the potential to contribute to the mapping of DBS effects-related electrophysiological or metabolic markers in spatial dimensions.

## Conclusions

Translational studies using human-mimicking models are pivotal for exploring electrophysiological and chemical markers for aDBS, advancing technologies, devices, and programming, and investigating the mechanisms underlying various DBS approaches. Concurrently, animal studies provide the flexibility to examine symptom-specific neural oscillations and cell populations without spatial or temporal limitations. Using optogenetic stimulation, we can identify specific cell types and their anatomical fiber connections responsible for symptomatic improvement, enabling the optimization of DBS parameters and electrode placement^9^. Taking advantage of the vector-based A53T-α-Syn PD model^111,151–153^, which mirrors the progressive nature and full course of the disease, we can address the best timing for DBS initiation to achieve a disease-modifying effect. Moreover, the pathological markers across different disease stages that can predict long-term DBS outcomes can also be explored through basic science. Finally, we indicate the inherent limitations across species and emphasize the importance of mutual translation between human and animal models. This reciprocal approach is crucial for identifying shared neuromodulation mechanisms across species, facilitating the translation of mechanistic insights from models to patients.
