# Author Correction: A framework for translational therapy development in deep brain stimulation

**DOI:** 10.1038/s41531-025-00890-8

**Published:** 2025-03-04

**Authors:** Jiazhi Chen, Jens Volkmann, Chi Wang Ip

**Affiliations:** Department of Neurology, University Hospital of Würzburg, Josef-Schneider-Straße 11, Würzburg, Germany

**Keywords:** Motor control, Diseases of the nervous system, Cellular neuroscience

Correction to: *npj Parkinson’s Disease* 10.1038/s41531-024-00829-5, published online 08 November 2024

In this article the author name Jiazhi Chen was incorrectly written as Jia Zhi Chen. The original article has been corrected.

